# The quagga mussel genome and the evolution of freshwater tolerance

**DOI:** 10.1093/dnares/dsz019

**Published:** 2019-08-29

**Authors:** Andrew D Calcino, André Luiz de Oliveira, Oleg Simakov, Thomas Schwaha, Elisabeth Zieger, Tim Wollesen, Andreas Wanninger

**Affiliations:** 1 Department of Integrative Zoology, University of Vienna, Vienna, Austria; 2 Department of Molecular Evolution and Development, University of Vienna, Vienna, Austria; 3 Developmental Biology Unit, European Molecular Biology Laboratory, Heidelberg, Germany

**Keywords:** *Dreissena*, osmoregulation, genome, quagga, aquaporin

## Abstract

Freshwater dreissenid mussels evolved from marine ancestors during the Miocene ∼30 million years ago and today include some of the most successful and destructive invasive species of freshwater environments. Here, we sequenced the genome of the quagga mussel *Dreissena rostriformis* to identify adaptations involved in embryonic osmoregulation. We provide evidence that a lophotrochozoan-specific aquaporin water channel, a vacuolar ATPase subunit and a sodium/hydrogen exchanger are involved in osmoregulation throughout early cleavage, during which time large intercellular fluid-filled ‘cleavage cavities’ repeatedly form, coalesce and collapse, expelling excess water to the exterior. Independent expansions of aquaporins coinciding with at least five freshwater colonization events confirm their role in freshwater adaptation. Repeated aquaporin expansions and the evolution of membrane-bound fluid-filled osmoregulatory structures in diverse freshwater taxa point to a fundamental principle guiding the evolution of freshwater tolerance and provide a framework for future species control efforts.

## 1. Introduction

Molluscs evolved in the ocean, yet today there are ∼5,000 freshwater species worldwide originating from >40 independent colonization events.^1–3^ While most freshwater mollusc species are gastropods (ca. 4,000 species),^2^ several bivalves (e.g. *Dreissena rostriformis*, *Dreissena polymorpha*, *Limnoperna fortunei* and *Corbicula fluminea*) have proven to be highly successful and ecologically disruptive invasive species that function as ecosystem engineers of colonized environments.^4–7^

**Figure 1:**
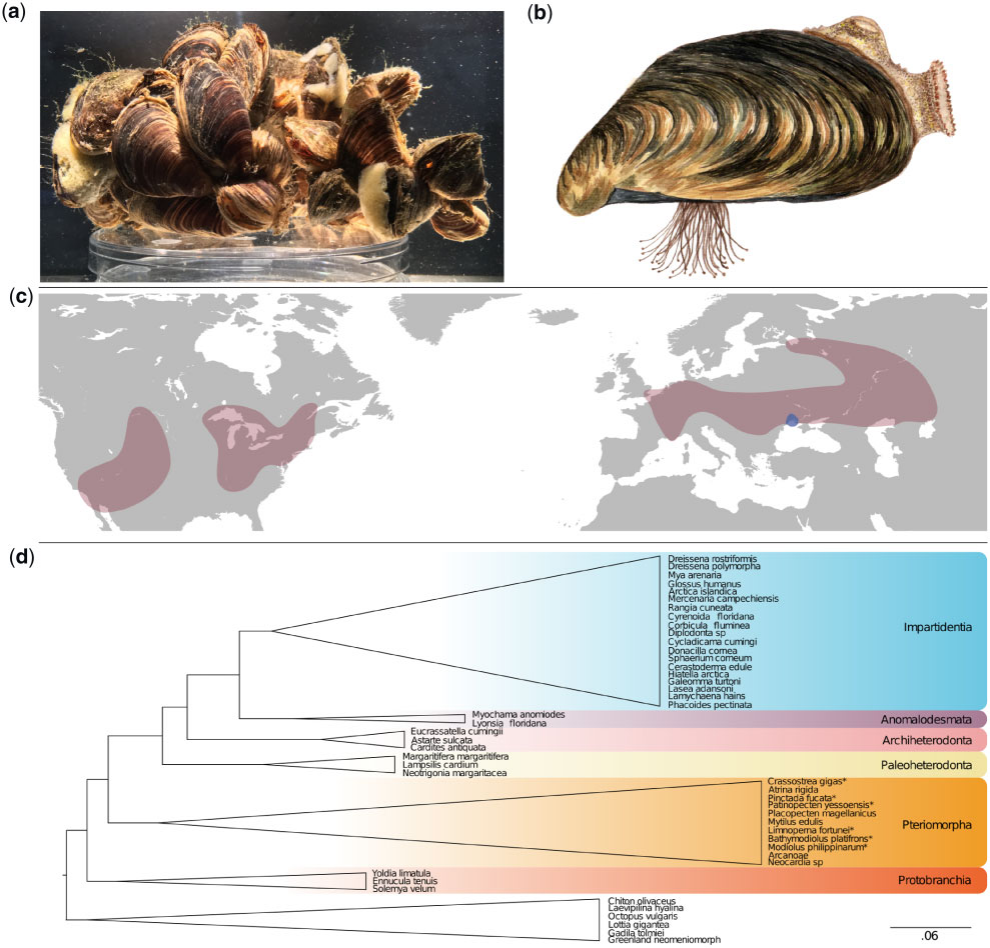
The quagga mussel, *D. rostriformis*. (a) Quagga mussels form dense aggregations connected with strong byssal threads. Aggregates are often associated with other benthic species such as sponges. (b) Illustration of a single quagga mussel demonstrating the distinct banding pattern of the shell and the dense clump of byssus threads that enables them to adhere to both natural and manufactured substrates. (c) Global distribution of the quagga mussel highlighting native (blue) and colonized (red) habitats. (d) Condensed phylogeny of Bivalvia^51^ based on a supermatrix composed of 47 molluscan taxa covering 1,377 orthogroups. The quagga mussel is positioned amongst the Imparidentia. Species with sequenced genomes are marked with a *. All nodes possess Shimodaira–Hasegawa (SH) test support values equal to 1. Color figures are available at *DNARES* online.

Bivalves have invaded freshwater habitats on at least 11 occasions.^3^^,^^8^ Despite the independence of these events, freshwater species all typically maintain their internal osmotic pressure above that of their environment.^9^^,^^10^ This differs to marine and brackish water species which typically maintain their body fluid osmolarity at equilibrium with their environment.^11^ Due to their large surface area to volume ratio, the issue of cellular osmoregulation is expected to be particularly acute for the eggs and embryos of broadcast spawners such as the quagga mussel *D. rostriformis* (Fig. 1a and b). The ability of quagga mussels to withstand the harsh osmotic conditions of the freshwater environment during embryogenesis may contribute to their capacity for rapid spread through newly colonized habitats.

Quagga mussels evolved in the isolated Lake Pannon between 10 and 8.5 million years ago.^12^ Their native range today spans the Black and Caspian Sea catchments which are remnants of this ancient lake. Invasive populations of quagga mussels are now established throughout mainland Europe, the British Isles and North America where they have caused enormous ecological and economic impacts^13–16^ (Fig. 1c). Quagga mussels, like their close relatives the zebra mussels (Fig. 1d), have the capacity for high density biofouling and for nutrient redistribution of colonized habitats.^17^ Furthermore, in invaded habitats once dominated by zebra mussels, the introduction of quagga mussels has repeatedly lead to the replacement of the former with the latter.^18^ This is most well illustrated in the great lakes of North America where nearly all of the area once colonized by zebra mussels has since been overtaken by quagga mussels.^19^^,^^20^

A conspicuous feature of the early cleavage stages of some freshwater and terrestrial molluscs is the formation of a large, lens-shaped, fluid-filled cavity between dividing cells.^21^^,^^22^ Following cell division, small cavities appear along the cell–cell border, gradually coalescing until a single large cavity remains. The contents of the cleavage cavity are then rapidly discharged to the environment before a new cavity begins to form. An osmoregulatory role for cleavage cavities has been previously suggested but their patchy phylogenetic distribution indicates a complex evolutionary history.^21^^,^^23^^,^^24^

To identify genetic signatures underpinning the capacity of quagga mussels for freshwater tolerance, we sequenced, assembled and annotated the ∼1.6 gigabase (Gb) genome of *D. rostriformis*. Using both developmental and comparative approaches, we reveal the machinery and structures utilized by quagga mussels for embryonic osmoregulation, which is a key feature underpinning their invasive capacity for rapid range expansion. Furthermore, we demonstrate that these innovations have also independently evolved on multiple occasions coinciding with freshwater colonization events by a diverse range of species.

## 2. Materials and methods

A detailed Materials and methods section can be found in Supplementary Materials. A brief overview follows here.

### 2.1. Sampling and sequencing

A single male *D. rostriformis* selected for DNA extraction was collected from the Danube river in Vienna, Austria (48°14′45.9″N, 16°23′38.0″E) and treated with an antibiotic–antimycotic solution (Gibco 15240062) to minimize the risk of bacterial or fungal contamination (Supplementary Material SM 1). Three shotgun libraries including two 300-bp insert and one 550-bp insert polymerase chain reaction free library were prepared in addition to three long jumping distance libraries (3, 8 and 20 kb) which were sequenced on an Illumina HiSeq 2500. Four developmental RNA-seq libraries (gastrula, trochophore, early veliger and juvenile) were sequenced on an Illumina HiSeq 2500 for transcriptome construction and genome annotation. An additional 18 RNA-seq libraries were also sequenced for the purpose of quantitative gene expression analyses. Proprietary read processing of the long jumping distance libraries including quality and adaptor trimming was performed by Eurofins Genomics. Quality and adaptor trimming of the shotgun libraries was performed with trimmomatic^25^ and library quality assessed with FastQC (www.bioinformatics.babraham.ac.uk/projects/fastqc/). Kmer counting was performed with Jellyfish^26^ and genome size and heterozygosity were estimated with GenomeScope.^27^

### 2.2. Genome assembly

Genome contig assembly, scaffolding and gap-closing were performed with Platanus^28^ on the SGI Altix Ultra Violet 1000 located at the Johannes Kepler University, Linz, Austria (Supplementary Material SM 2). Following assembly, heterozygosity was reduced using the Redundans pipeline^29^ performed on the Life Science Compute Cluster (CUBE) located at the University of Vienna, Austria. Quast^30^ was used to assess genome assembly metrics and read re-mapping was performed with Bowtie2.^31^ Potential contamination of the assembly was assessed with BlobTools.^32^

### 2.3. Genome annotation

A custom RepeatModeler^33^ library was built and used to mask repetitive elements in the genome assembly with RepeatMasker^34^ (Supplementary Material SM 3). *Ab initio* gene prediction was performed with Augustus^35^ and SNAP.^36^ Homology-based prediction was performed with GeneWise based on the alignment of the complete annotated protein coding transcriptomes of five species (*Crassostrea gigas*, *Octopus bimaculoides*, *Lottia gigantea*, *Lingula anatina* and *Drosophila melanogaster*) to the *Dreissena* genome with TBLASTN (*e* value ≤ 1e-5) to produce accurate spliced alignments. *De novo* transcriptome assemblies from the four developmental RNA-seq libraries were produced with Binpacker^37^ using five different kmer values (k23, k25, k27, k29 and k32) which were subsequently merged with Velvet^38^ and de-duplicated with Dedupe.^39^ Open reading frames were predicted with Transdecoder^40^ and these putative protein coding transcripts were mapped back to the genome assembly with GMAP.^41^ For reference-based transcriptome assembly, the trimmed RNA-seq libraries were mapped against the genome assembly with STAR aligner^42^ and then assembled with StringTie.^43^ These assemblies were merged with the StringTie merge function and open reading frames predicted with Transdecoder. The *de novo* transcriptome assembly, the reference-based transcriptome assembly, the two *ab initio* gene prediction outputs and the homology-based gene prediction were used as input for EvidenceModeler.^44^ The resulting set of transcripts were filtered to include those that have homology to either the Pfam, uniref^90^ or CDD databases or for which there is evidence of expression in one of the 22 developmental RNA-seq databases. Gene models that overlapped with repetitive sequences as assessed by RepeatMasker for at least 50% of their length were also excluded. Final transcriptome ‘completeness’ was assessed with BUSCO.^45^

### 2.4. Phylogenetics and phylogenomics

**Figure 2:**
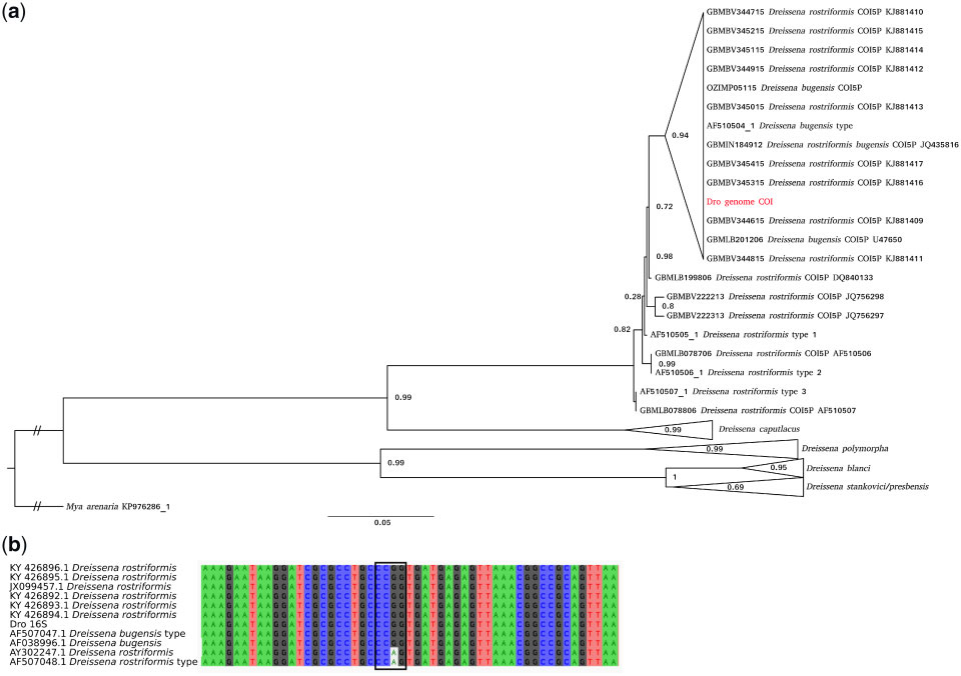
Dreissenid *COI* phylogeny and 16S alignment. (a) The sequence in red is the *COI* from the genome sequenced here, sequences with ‘type’ in the name were obtained by Therriault et al.^49^ and the remaining sequences were obtained from the BoLD database. *Dreissena stankovici* and *D. presbensis* are likely to represent a single species called *D. carinata* (Dunker, 1853). SH test support values are indicated. (b) Multiple sequence alignment of *16S* rRNA. The *16S* rRNA from the genome sequenced here is named *Dro 16S*, sequences with ‘type’ in the name were obtained by Therriault et al.^49^ and the remaining sequences were downloaded from NCBI. The box highlights a motif identified by Therriault et al.^49^ as diagnostic for discerning *bugensis* (CCGG) from other *D. rostriformis* clades (CCAG). Color figures are available at *DNARES* online.

To determine the taxonomic status of the quagga mussel sampled here, a phylogeny was produced using FastTree 2^46^ with the LG evolutionary model as determined by ProtTest 3^47^ based on cytochrome c oxidase subunit I (*COI)* sequences using all dreissenids available from Barcode of Life Database (BoLD),^48^ in addition to those used in a previous taxonomic analyses of the family^49^ (Fig. 2a). An alignment using MAFFT^50^ of 16S sequences from Therriault et al.,^49^ from NCBI and from our assembly was also undertaken to determine the subspecies sampled (Fig. 2b). A phylogenomic tree was produced to confirm the position of the quagga mussel within the Bivalvia (Supplementary Material SM 4). A total of 1,377 curated orthogroups obtained from 40 molluscan taxa, including 34 bivalves^51^ were downloaded and used to build profile-hidden Markov models (pHMMs) and multiple sequence alignments to extend the orthologue groups using HaMStr.^52^ Multiple sequence alignments were generated with MAFFT^50^ and pHMMs built with hmmbuild from the HMMER3 package.^53^ The extended orthogroups were concatenated into a super matrix with FASconCAT^54^ and the phylogeny inferred with FastTree 2.

### 2.5. Identification and phylogenetic analysis of embryonic osmoregulatory candidate genes

The genes required for early embryonic osmoregulation are likely to be maternally inherited and so their relative expression levels are unlikely to be affected by the osmotic environment of the embryo. As such, an approach was required that did not rely on changes to relative transcript abundance under experimental osmotic conditions, such as differential gene expression analysis. To identify genes encoding proteins with known roles in osmoregulation, ionic homeostasis and excretion, the full set of *Dreissena* gene models were used to search the KEGG database^55^ using the KAAS search tool.^56^ Genes encoding transmembrane proteins involved in one of five KEGG pathways: (i) vasopressin-regulated water reabsorption, (ii) proximal-tubule bicarbonate reclamation, (iii) collecting duct acid secretion, (iv) aldosterone-regulated sodium reabsorption and (v) endocrine and other factor calcium reabsorption, were retained (Supplementary Material SM 5). To determine which of the candidate genes are significantly up-regulated during early embryogenesis, RNA-seq reads from the 18 developmental libraries were pseudoaligned to the *Dreissena* transcriptome with Kallisto^57^ to determine the normalized Transcripts Per Million (TPM) value for each gene at each developmental time point. This pipeline was repeated with equivalent data from the marine species *C. gigas*. A Fisher’s exact test was performed in R to identify those candidate genes that are up-regulated during the non-swimming early developmental stages (unfertilized eggs, 2–4 cell embryos and gastrulas) against the background, defined as the average TPM value of all remaining developmental stages, using an *e* value cut-off of 1e-6. Any gene that met this criteria in both *Dreissena* and *Crassostrea* was removed from the list of candidates while those uniquely up-regulated in *Dreissena* during early development were retained. While a marine species more closely related *Dreissena* would have made a more desirable comparative target than *Crassostrea*, at the time of this project’s undertaking no such high-resolution developmental RNA-seq datasets were publicly available for any other marine bivalve. To place the candidate genes in a phylogenetic context, family members were obtained from related species using Pfam hidden Markov models with hmmsearch from the HMMER package.^53^ Amino acid sequences were aligned with MAFFT,^50^ visualized with Aliview^58^ and trimmed with BMGE.^59^ Tree construction was performed with FastTree 2^46^ using the LG evolutionary model as determined by ProtTest 3^47^ for all alignments (Supplementary Material SM 6).

### 2.6. 3D Structural protein modelling

The aquaporin orthologue found to be highly expressed during early *Dreissena* embryogenesis (see Embryonic osmoregulatory gene identification and characterization) was uploaded to SWISS-MODEL^60^ for structural modelling (Supplementary Material SM 7). Models were built from the top 14 templates (as determined by a quaternary structure quality estimate of >0.5), which included structures from four aquaporin orthologues—*AQPO* (PDB: 1YMG), *AQP1* (PDB: 5C5X), *AQP4* (PDB: 1J4N) and *AQP5* (PDB: 2ZZ9). Stabilization of the quaternary structure through the formation of salt bridges was predicted with ESBRI,^61^ protein structure visualizations were performed with PyMOL^62^ and peptide logos were constructed with Skylign.^63^

### 2.7. Embryogenesis under osmolarity challenges

Adult mussels collected from the Danube River in Vienna, Austria, were induced to spawn through immersion in 0.5 mM serotonin dissolved in filtered river water (FRW). The jelly layer of the eggs was removed by immersion in a pH 8.6 FRW solution before being washed in ambient FRW (Supplementary Material SM 8). De-jellied eggs were fertilized through the introduction of sperm and these were subsequently washed and transferred to a WillCo glass bottom dish for observation on a Leitz Labovert inverted microscope (Leica Microsystems, Wetzlar, Germany). Ambient conditions meant that development occurred at ∼26°C. To test the impact of osmolarity on embryogenesis, a cohort of de-jellied, fertilized embryos were allowed to develop in a high osmolarity solution consisting of an artificial seawater salt mix (Sera Marin, 1.75 ppt) dissolved in FRW. Another cohort was allowed to develop in a low osmolarity solution consisting of a dilution of FRW in reverse-osmosis filtered water (1:3 dilution).

## 3. Results

### 3.1. Genome sequencing and assembly

In total, 147.8 Gb of short-read shotgun sequence was generated, equating to ∼92-fold coverage of the estimated 1.6-Gb quagga mussel genome. Kmer assessment with GenomeScope indicated a high heterozygosity rate of 2.4% which is a well-publicized hindrance to contiguous genome assembly and was the primary reason behind the decision to use Platanus^28^ for the genome assembly. Contig assembly, scaffolding and gap-closing, and haplotype reduction with Redundans,^29^ resulted in an assembly covering 1.24 Gb with an N50 of 131.4 kb. The difference of ∼360 Mb between the assembled genome and the predicted genome size is most likely explained by the collapsing or reduction of highly repetitive regions, as has been observed with other genome assemblies.^64^ Mapping of the paired end libraries back to the completed genome resulted in 94.5% realignment, confirming the integrity of the assembly. RepeatMasker revealed a repeat population covering 31.9% of the assembly with most bases (24.2%) masked by unclassified repeats (Supplementary Material SM 3).

BlobTools^32^ was used to search for scaffolds in the final assembly with likely non-target origins. This demonstrated no evidence of contamination (Supplementary Material SM 2). Quagga mussels live in dense communities in close association with a high diversity of bacterial, metazoan and other eukaryotic species (Fig. 1a). The absence of contaminating scaffolds in the genome assembly is most likely the result of the thorough efforts employed to avoid contamination prior to DNA extraction.

### 3.2. Genome annotation and transcriptome construction

The pipeline employed to construct the complete set of gene models from the quagga mussel genome incorporated *ab initio*, homology, *de novo* transcriptome and reference-based transcriptome gene models (Supplementary Material SM 3). After processing these various inputs with EvidenceModeler,^44^ 99,522 gene models were determined. Further filtering for genes that had homology to entries in one of three publicly maintained databases (see Materials and methods) had evidence of expression in at least one RNA-seq dataset and did not overlap with repeat elements for >50% of its length resulted in a set of 37,681 coding genes models. These included 95% of either full length or fragmented metazoan BUSCO v2.0 genes^45^ (Supplementary Material SM 3). Homology support for the 37,681 coding genes models ranged from 68% (CDD) to 86% (uniref90) with a total 87% of gene models showing homology support from at least one of the three databases examined.

### 3.3. Quagga mussel phylogeny

The taxonomic nomenclature of the quagga mussel is unresolved. The two most common assignments for this species are *D. rostriformis* and *Dreissena bugensis* with *rostriformis* often designated as a subspecies of the latter.^49^^,^^65^ Reported differences between *D. rostriformis* and *D. bugensis* include the depth at which they are found and the salinity of their native habitats, however attempts to discriminate the two species on the basis of morphology have proven difficult due to the high level of intraspecific relative to interspecific variation.

Our results support the discontinuation of *D. bugensis* as a species distinct from *D. rostriformis*.^66^ They also indicate that the species names allocated to the quagga mussel samples in the BoLD database (*D. rostriformis*, *D. bugensis*, *D. rostriformis bugensis*) do not represent distinct genetic clades. As such, the preferred name is *D. rostriformis* as it is the oldest of the candidates (*D. rostriformis*, ANDRUSOV 1839 versus *D. bugensis*, ANDRUSOV 1867). A single well-supported clade within the *D. rostriformis* branch that includes the sample sequenced here, in addition to the *D. bugensis* sample collected by Therriault et al.^49^ was identified, suggesting that the shallow freshwater form may represent a genetically distinct group, although more dedicated sampling will be required to confirm this. This analysis was unable to resolve the distinction between the BoLD *D. presbensis* and *Dreissena stankovici COI* sequences. Neither of these species names is marked as ‘accepted’ on the World Register of Marine Species (WoRMS) database^67^ and it is likely that both are synonyms for *Dreissena carinata* (Dunker, 1853).

In examining the *16S* rRNA sequences of *D. rostriformis* and *D. bugensis*, Therriault et al.^49^ identified a single nucleotide difference between the two forms which could be used as a diagnostic identification tool by cleaving polymerase chain reaction products with the restriction enzymes *Msp*I or *Hpa*II. The *16S* sequence from the sample sequenced here is consistent with *D. rostriformis*, however, as with the COI analysis, more dedicated sampling will be required to confirm this as a diagnostic feature (Fig. 2b).

Phylogenetic assessment placed the quagga mussel amongst the Imparidentia, sister to the congeneric zebra mussel *D. polymorpha* (Supplementary Material SM 4). Consistent with previous reports, we find that the closest relative to the *Dreissena* lineage is the soft shell clam *Mya arenaria*^51^ and that this group has the longest branch length of the Imparidentia.

### 3.4. Embryonic osmoregulatory gene identification and characterization

**Figure 3:**
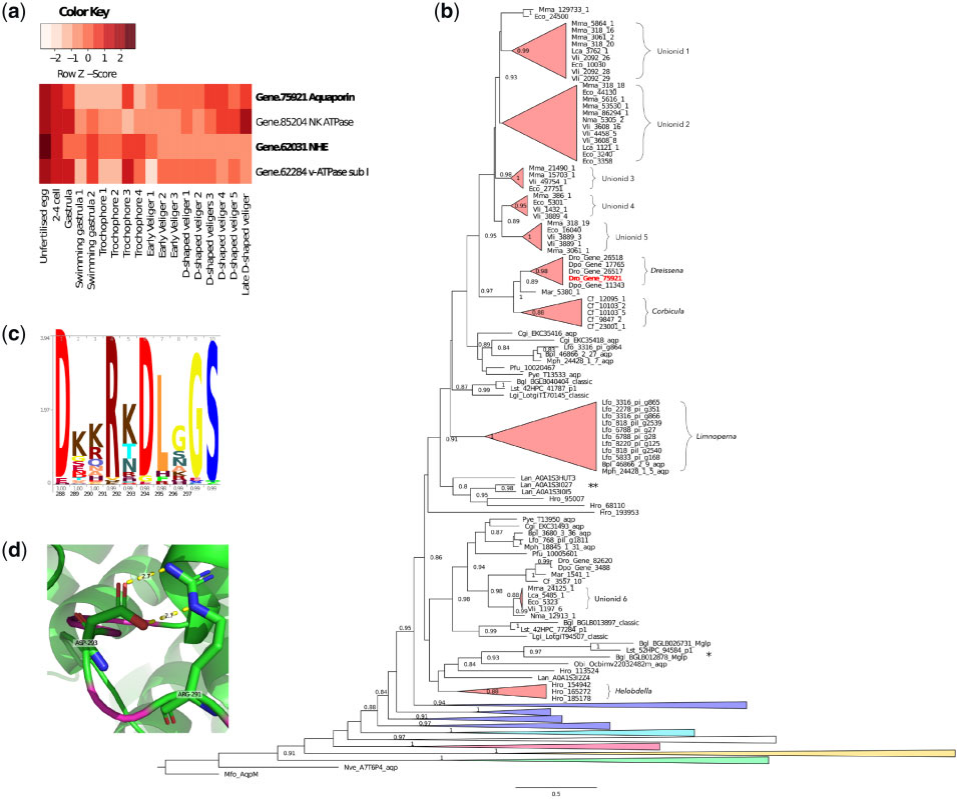
Embryonically expressed osmoregulatory genes. (a) Heat map of expression of candidate osmoregulatory genes highly expressed during embryogenesis prior to the free-swimming stage. Three genes (highlighted in bold) are highly expressed in *Dreissena* but not in similar stages of the marine oyster *Cr. gigas* (see Supplementary Material SM 6.2). (b) Phylogenetic tree of aquaporins with emphasis on classical lophotrochoaquaporins (red) with other classes (green—aquaglyceroporins, yellow—unorthodox, pink—EGLPs, white—undescribed annelid/brachiopod clade, light blue—aquaamoniaporins, blue—classical aquaporins) collapsed. Note the independent expansions associated with the freshwater lineages *Dreissena*, *Corbicula*, *Limnoperna*, the unionid mussels and the annelid leech *Helobdella*. * indicates a clade of long branch freshwater gastropod sequences previously annotated as malacoglyceroporins. ** indicates an expanded clade of marine brachiopod sequences. SH support values under 0.8 are not shown. (c) Peptide logo of the highly charged lophotrochoaquaporin loop D with occupancy and amino acid position indicated on the *x*-axis, respectively. (d) Predicted structure of the *Dreissena* lophotrochoaquaporin *Dro.75921* loop D (magenta) wrapped to *Bos taurus AQP1* (PDB: 1j4n.1) showing the predicted salt bridge formed between Arg-291 and Asp-293. Color figures are available at *DNARES* online.

In total, 27 aquaporins (Pfam: PF00230.19), 8 sodium/potassium ATPases (Pfam: PF00287.17), 13 sodium/hydrogen exchangers (NHE, Pfam: PF00999.20), 8 hydrogen/carbonate co-transporters (Pfam: PF00955.20), 12 voltage gated chloride channels (PF00654.19), 9 cation ATPases (Pfam: PF00689.20, PF13246.5 and PF00690.25) and 17 hydrogen ATPases (Pfam: PF00006.24, PF02874.22, PF01813.16, PF03223.14, PF03179.14, PF01496.18, PF01992.15 and PF01991.17) were identified from the quagga mussel gene models, while in *Crassostrea*, 2 sodium/potassium ATPases, 11 NHEs, 8 hydrogen/carbonate co-transporters, 6 voltage gated chloride channels, 10 cation ATPases and 11 hydrogen ATPases (Supplementary Material SM 5) were found. Early embryogenesis (pre-swimming stages that lack distinct osmoregulatory organs) in the quagga mussel is associated with significantly (1e-6) high expression levels of an aquaporin (Gene.75921), an NHE (Gene.62031), a vacuolar ATPase subunit a (Gene.62284) and a sodium potassium ATPase (Gene.85204) while in *Crassostrea*, the expression profiles of two sodium/potassium ATPases (EKC41758, EKC32470) and one cation ATPase (EKC34610) were highly correlated with similar stages of development. High expression levels of aquaporins, vacuolar ATPase (v-ATPase) subunit as or sodium/hydrogen exchangers were not observed in the pre-swimming embryonic stages of *Crassostrea* (Fig. 3a).

A phylogeny of 291 aquaporin genes including 168 bivalve sequences from 15 species spanning both marine and freshwater taxa successfully resolved the 4 major animal aquaporin classes—classical aquaporins, aquaamoniaporins, unorthodox aquaporins and aquaglyceroporins^68^ (Fig. 3b;Supplementary Material SM 6). Entomoglyceroporins (EGLPs),^69^ malacoaquaporins,^70^*Drosophila* intrinsic proteins (DRIPs)^71^ and *Pyrocoelia rufa* integral proteins (PRIPs)^72^ were also resolved. In addition, a group consisting of representatives from the brachiopod *Lingula* and the freshwater annelid leech *Helobdella robusta* were found to branch between the aquaammoniaporins and the EGLPs. No support for the malacoglyceroporin (Mglp) clade was found in our study.^70^^,^^73^

A previously unidentified class of lophotrochozoan-specific classical aquaporins (hereafter referred to as lophotrochoaquaporins) that includes the embryonically highly expressed *Dreissena* orthologue, appears to have expanded on at least four occasions, coinciding with the freshwater bivalves *Dreissena*, *C. fluminea*, *L. fortunei* and the Unionidae (a species-rich monophyletic taxon of freshwater paleoheterodont bivalves). In contrast, none of the marine bivalves shows evidence of such an expansion. Outside of the bivalves, *Helobdella* also has an expanded set of lophotrochoaquaporins while the freshwater gastropods *Lymnaea stagnalis* and *Biomphalaria glabrata* possess long branch lengths without evidence of expansions (Fig. 3b).

In the quagga mussel, three lophotrochoaquaporin orthologues form a clade with those of the congeneric freshwater *D. polymorpha* (two orthologues) and the closely related marine species *M. arenaria* (one orthologue). While the five orthologues of the freshwater *C. fluminea* were not annotated against a genome, the level of sequence divergence between the orthologues makes it likely that at least three represent true paralogues (Supplementary Material SM 6), the same number found in the quagga mussel.

The distantly related freshwater golden mussel *L. fortunei* possesses an expanded set of nine lophotrochoaquaporin orthologues whereas its closest relatives, the marine *Modiolus philippinarum* and *Bathymodiolus platifrons*, possess only a single copy each. No genomic resources are yet available for any of the freshwater paleoheterodonts (unionids) and thus, to avoid conflating transcript variants with true orthologues, expanded aquaporin clades were only annotated if they included representatives from at least three species. Four unionid clades were thus identified, indicating that the last common ancestor of these species likely already possessed an expanded repertoire of lophotrochoaquaporins. Only a single lophotrochoaquaporin orthologue was identified in the marine paleoheterodont *Neotrigonia. margaritacea*, which is the closest marine relative of the freshwater unionids. While the identification of more *N. margaritacea* orthologues with increased sampling cannot be ruled out, it appears that the paleoheterodont lophotrochoaquaporin expansion occurred after the divergence of the marine and freshwater species and before speciation of the unionids.

The v-ATPase subunit a is the most diverse of the v-ATPase subunits^74^^,^^75^ and is responsible for targeting the v-ATPase complex to specific sites within the cell.^76^^,^^77^ No comprehensive phylogeny of metazoan v-ATPase subunit a sequences is yet available. In vertebrates, four subunit a isoforms have been identified, each with distinct functions and expression patterns.^78^^,^^79^ It is unknown how these relate to the v-ATPase subunit a isoforms of other metazoan lineages. We find v-ATPase subunit a expansions in each phylum investigated, however molluscs appear to have undergone two rounds of diversification giving rise to two distinct monophyletic subclasses (Supplementary Material SM 6).

Sodium hydrogen exchangers form part of the monovalent cation proton antiporter (CAP) superfamily.^80^ Phylogenetic analysis successfully resolved all previously reported animal CAP classes—NHA, PM-NHE and Endo/TGN IC-NHE and NHE8-like IC-NHE (Supplementary Material SM 6). We were also able to resolve the position of the enigmatic mammalian sperm NHEs with a well-supported clade consisting of deuterostome, lophotrochozoan and ecdysozoan orthologues, in addition to the plant SOS1 sequences. The non-animal clades CHX, NhaP and plant vacuolar were also resolved. We also found support for two previously unreported animal CAP clades. The first, consisting of deuterostome and lophotrochozoan sequences, is most closely aligned to the plant CHX transporters. No molluscan sequences were identified from this clade. The second was a large lophotrochozoan-specific family of NHEs most closely aligned to the PM-NHEs found in all major animal superphyla. The quagga mussel NHE found to be highly expressed during early embryogenesis (Gene.62031) is a member of this lophotrochozoan-specific NHE family.

Protein structural modelling of the embryonically highly expressed *Dreissena* lophotrochoaquaporin orthologue (Dro.75921) with SWISS-MODEL^60^ showed that the most structurally variable regions as measured by the QMEAN score corresponded to loops A, C and E. The exception to this was the model-template alignment with *AQP1* which also showed strong structural similarity to *Dro.75921* through loop D with the QMEAN not dropping below 0.68 [SWISS-MODEL cut-off for low quality equals 0.6 (Supplementary Material SM 7)]. Lens fibre major intrinsic protein, *AQP4* and *AQP5* have minimum QMEAN scores through loop D of 0.48, 0.48 and 0.58, respectively. Amino acid conservation was considerably higher for the Loop D region of lophotrochoaquaporins than for other classical aquaporins such as AQP4 (Fig. 3c;Supplementary Material SM 7). Loop D amino acids Arg-291 and Asp-293 of Dro.75921 were also predicted to form a salt bridge which acts to stabilize quaternary structure (Fig. 3d; Supplementary Material SM 7).

### 3.5. Embryonic response to osmolarity challenges

**Figure 4:**
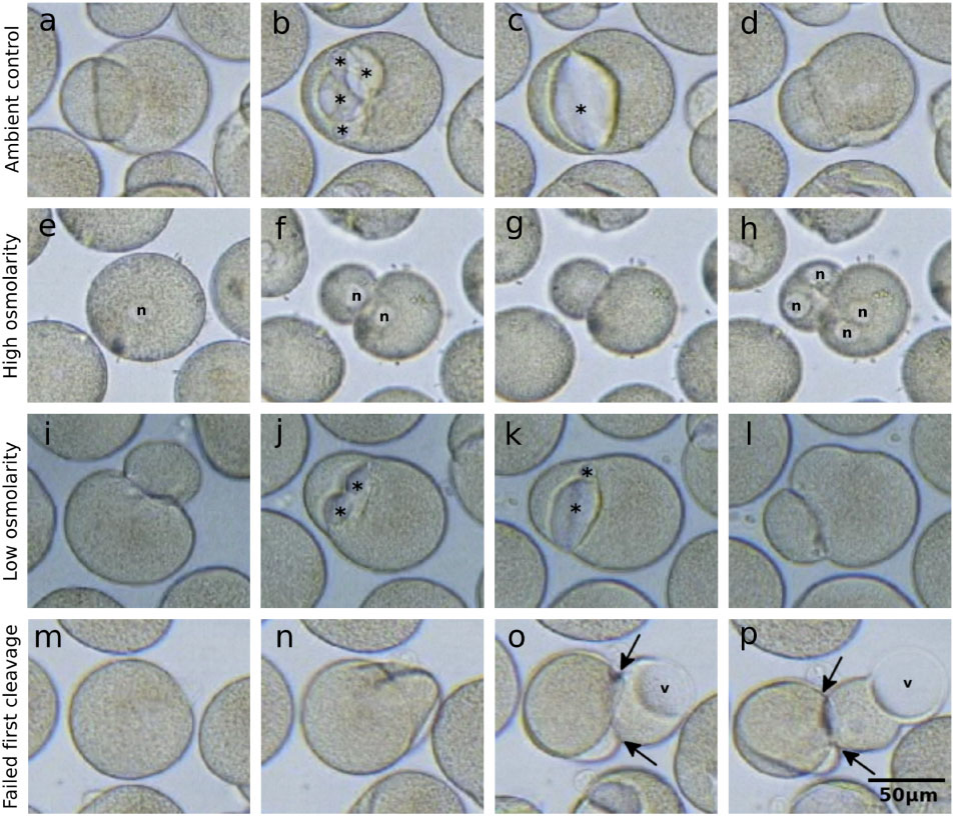
Cleavage cavities in developing *Dreissena* embryos. (a–d) Formation of cleavage cavities during the first embryonic cleavage under ambient conditions. (e–h) First and second embryonic cleavages under high salinity demonstrating the lack of cleavage cavity formation. (i–l) Formation of cleavage cavities during the first embryonic cleavage under low salinity. (m–p) Failed first cleavage leading to rupture of the fertilization envelope and the extrusion of an amoeboid projection with a large vacuole. * indicates cleavage cavities, N indicates nuclei, v indicates intracellular vacuoles and arrows indicate fertilization envelope ruptures. Color figures are available at *DNARES* online.

In both control (ambient osmolarity) and treated (low and high osmolarity), first cleavage was observed to occur at ∼1 hpf with the second and third cleavage occurring at ∼1-h intervals. No impact on fertilization or development as a consequence of jelly coat removal was observed. The first cleavage cavity began to form between the two daughter cells shortly after the first cleavage was completed (Fig. 4a–d and Supplementary Material SM 8 and Supplementary Movie S1). As has been observed in several species, cleavage cavity formation begins simultaneously at several positions along the cell–cell margin, resulting in small lens-shaped fluid-filled cavities, which gradually grow and coalesce until a single cavity remains. Cleavage cavities often grow to occupy a substantial proportion of the total embryo volume, leaving only a small ring traversing the circumference of the embryo where cell–cell contact remains intact. Eventually, this final ring of contact is breached by the growing cavity whereupon the fluid in the cavity is rapidly discharged, possibly through the release of tension built up in the fertilization envelope. This discharge results in the collapse of the cavity.

The process of cleavage cavity inflation and collapse typically repeats two to four times during each of the first three cleavages. At the eight-cell stage, a central cavity forms and this also appears to periodically inflate and collapse in a similar way to the cleavage cavities (Supplementary Material SM 8). Over the course of observation, most embryos remained at the approximate volume at which they began, notwithstanding the repeated inflation and collapse attributable to cleavage cavity activity. In contrast, those eggs that failed to become fertilized gradually increased in volume over the recording period.

In contrast to embryos raised under ambient osmolarity, embryos raised in the high salt solution progressed through the cleavage stages either without the production of cleavage cavities, or, in a few cases, with highly reduced cleavage cavities (Fig. 4e–h; Supplementary Move S2). Zygotes were also observed to reduce in volume prior to the first cleavage before stabilizing (Supplementary Material S8). Embryos raised under low osmotic conditions developed normally and produced large cleavage cavities similar to those in ambient conditions (Fig. 4i–l and Supplementary Material SM 8 and Supplementary Movie S3).

On rare occasions under ambient osmotic conditions, cleavage of the embryo fails (Fig. 4m–p; Supplementary Movie S1). We have observed that embryos that are successfully fertilized, as determined by the presence of polar bodies, but fail to cleave correctly, are at an increased risk of fertilization envelope rupture. When the fertilization envelope ruptures, part of the embryo is extruded from the fertilization envelope, leading to the formation of large highly active amoeboid processes that often contain large vacuole-like structures (Supplementary Movie S1).

## 4. Discussion

Through the sequencing and annotation of the genome of the invasive quagga mussel, we have identified key machinery underlying the capacity for *Dreissena* to osmoregulate under hypo-osmotic conditions from as early as the two-cell stage of development. The capacity to regulate intercellular osmolarity during embryogenesis was a key step in the evolution of freshwater tolerance in dreissenids and has likely been pivotal to their success as invasive species.

High repeat content and heterozygosity levels both pose substantial challenges to contiguous genome assembly and so necessitate appropriate assembly pipelines. These parameters were key to the selection of Platanus^28^ and Redundans^29^ as the primary components of the *Dreissena* genome assembly workflow. The resulting annotated assembly represents one of the most complete bivalve genome assemblies available as measured by BUSCO^45^ completeness and DNA-seq read re-mapping (Supplementary Material SM 3). The difference of ∼360 Mb between the assembled genome (1.24 Gb) and the predicted genome size (∼1.6 Gb) is most likely explained by the collapsing or reduction of highly repetitive regions, as has been observed with other genome assemblies.^64^

In order to maintain a body osmolarity above that of the surrounding medium, organisms require mechanisms to either prevent the osmotic influx of water that would otherwise diffuse across membranes, or to excrete excess water that passes into their cells. Likewise, the efflux of small solutes (e.g. Na^+^, K^+^ and Cl^−^) that would otherwise be lost to the environment must be prevented or balanced with uptake to avoid deteriorating osmotic conditions.

Many freshwater protozoans are comparable in size to *Dreissena* embryos and they too require mechanisms to cope with the osmotic pressures of freshwater environments in the absence of complex multicellular excretory organs. Protozoan contractile vacuoles, like lophotrochozoan cleavage cavities, are membrane-bound structures that gradually fill with excess cellular water before rapidly discharging it to the exterior.^81^^,^^82^ Additionally, both molluscan cleavage cavities and protozoan contractile vacuoles are associated with aquaporins and vacuolar ATPases.^83^

Aquaporins are a class of highly selective transmembrane passive water transporters that in animals fall into one of four clades (classical, aquaamoniaporins, aquaglyceroporins or unorthodox/S-aquaporins) based on their function and their capacity to transport specific solutes in addition to or instead of water.^68^ A fifth group specific to insects, the EGLPs, have been previously assigned to the classical aquaporins,^68^ however our phylogenetic analyses indicate they may represent a sister group. In humans, aquaporins are associated with a number of pathologies and are important components of the eye, blood–brain-barrier and kidney.^84^ In molluscs, the diversity and function of aquaporins are less well described although mollusc-specific aquaporin clades have been identified.^70^^,^^73^

*Dreissena*, *Corbicula* and *Limnoperna* represent independent invasions of freshwater habitats and all three have close marine relatives^8^^,^^85^ (Supplementary Material SM 4). The lineage-specific aquaporin duplications in these three species reflect this evolutionary history while the aquaporin expansion in the paleoheterodonts appears to have occurred after the divergence of the freshwater unionids from their marine ancestors, but before unionid speciation (Fig. 3b).

In addition to facilitating water transport across osmotic gradients, hydrostatic gradients may also influence the directional transport of water by aquaporin channels. In the absence of an osmotic pressure gradient, rat *AQP1* channels mediate transmembrane water transport across a hydrostatic pressure gradient^86^ while human *AQP1* expressed in *Xenopus* oocytes exhibits reversible gating dependent on the surface tension of the membrane.^87^ In plants, membrane tension-dependent gating of the grapevine aquaporin *TIP2;1* has also been observed under hypotonic conditions.^88^

In lophotrochoaquaporins, loop D is highly charged and appears to be highly conserved, with two of the most conserved amino acids (Arg-291 and Asp-293 in *Dro.75921*) also predicted to form a salt bridge^61^ (Fig. 3d). In addition to the conductance of water through individual subunit pores, human *AQP1* conducts Na^+^, K^+^ and Cs^+^ cations through the central tetrameric pore which is gated by the highly charged cytoplasmic loop D.^89–91^ Increasing evidence that water and ion conductance of aquaporins can be influenced by factors such as pH,^92–95^ membrane tension^87^ and hydrostatic pressure^86^ suggests that a complex set of dynamic, localized parameters may interact to influence aquaporin activity.

The sodium/hydrogen exchanger found to be highly expressed during *Dreissena* embryogenesis is a member of the plasma-membrane NHEs and thus is likely required for the recovery of sodium lost to the environment across the plasma membrane. Neither the v-ATPase subunits nor the PM-NHEs have undergone an evolutionary pattern of expansion akin to that of the aquaporins in which repeated colonization events of freshwater environments are associated with gene family expansions (Fig. 3b and Supplementary Material SM 6).

Cleavage cavity formation has been observed in a wide range of freshwater and terrestrial molluscs^21–24^^,^^96^^,^^97^ as well as in the freshwater annelid leech *Helobdella*.^98^^,^^99^ The freshwater bryozoan *Paludicella articulata* also appears to form a structures reminiscent of molluscan cleavage cavities.^100^ In vertebrates, rat hepatocyte couplets induced to secrete bile through the application of the choleretic dibutyryl cAMP rapidly shunt intracellular AQP8 to the membranes at the cell–cell interface.^101^ This induces the flow of water, forming a fluid-filled intercellular structure (the canaliculus) that also bears a striking resemblance to *Dreissena* cleavage cavities.

The co-occurrence of cleavage cavity formation with high aquaporin and v-ATPase subunit a expression levels indicates a role for these proteins in *Dreissena* embryonic osmoregulation. While v-ATPases are most well-known for the acidification of intracellular vesicles, their roles in the acidification of extracellular spaces are well established.^102^^,^^103^ It is also significant that the only v-ATPase subunit that is highly up-regulated during cleavage cavity formation is an orthologue of subunit a, as it is this subunit that is responsible for subcellular targeting of the entire v-ATPase complex.^92–95^

The repeated aquaporin expansions in freshwater lophotrochozoans and the importance of aquaporins and v-ATPases to protozoan osmoregulatory structures suggests that this molecular machinery may have been co-opted by both the lophotrochozoan cleavage cavity and the protozoan contractile vacuole excretory systems.

In protozoans, the mechanism of contractile vacuole contraction remains elusive. Contractile elements such as actin–myosin-based systems do not appear to be associated with contractile vacuoles, leading some authors to suggest that ‘contractile’ vacuoles are not actually contractile.^81^ A more recent hypothesis posits that tension built up in the membrane of the contractile vacuole may provide the force required for fluid discharge.^104^

In many animals, post-fertilization physicochemical modifications that transform the vitelline envelope into the fertilization envelope typically result in increased rigidity and stiffness.^105–107^ While such modifications have not been well described in molluscs, we observed that *Dreissena* embryos that fail to cleave or to form cleavage cavities under ambient conditions appear to be at increased risk of fertilization envelope rupture (Fig. 4m–p and Supplementary Movie S1). In contrast, unfertilized *Dreissena* eggs gradually increase in volume, presumably due to the osmotic influx of water, without such a marked increase in susceptibility to vitelline envelope rupture (Supplementary Material SM 8).

We suggest a mechanism for *Dreissena* cleavage cavity filling and discharge whereby the mechanical properties of the fertilization envelope restrict cell expansion in a functionally analogous manner to that of the cell wall of plants. Under such circumstances, an increased turgor pressure induced by the osmotic influx of water triggers the flow of water through the aquaporin channels down a hydrostatic pressure gradient into the developing cleavage cavity. The rapid expulsion of the contents of the cleavage cavity is thus mediated by tension stored in the fertilization envelope in a manner similar to the tension stored in the membranes of protozoan contractile vacuoles.^104^

The deployment of lophotrochoaquaporin water transport channels during early embryogenesis appears to have enabled *Dreissena* to regulate intercellular water levels as early as the two-cell stage. The ability of *Dreissena* embryos to excrete excess water via the formation of cleavage cavities is a function that in adult animals is usually reserved for complex organs such as nephridia or kidneys. It is likely that the evolution of this embryonic osmoregulatory mechanism involving aquaporin-mediated cleavage cavity formation was a crucial step in the adaptation of the quagga mussel—and likely many other aquatic animals—to freshwater environments.

## Supplementary Material

dsz019_Supplementary_Data
